# The role of ceRNA-mediated diagnosis and therapy in hepatocellular carcinoma

**DOI:** 10.1186/s41065-021-00208-7

**Published:** 2021-11-10

**Authors:** Yi Shi, Ji-Bin Liu, Jing Deng, Da-Zhi Zou, Jian-Jun Wu, Ya-Hong Cao, Jie Yin, Yu-Shui Ma, Fu Da, Wen Li

**Affiliations:** 1 College of Life Sciences and Chemistry, Hunan University of Technology, Zhuzhou, 412007 Hunan China; 2 Cancer Institute, Affiliated Tumor Hospital of Nantong University, Nantong, 226631 China; 3 National Engineering Laboratory for Deep Process of Rice and Byproducts, College of Food Science and Engineering, Central South University of Forestry and Technology, Changsha, 410004 Hunan China; 4 Department of Spine Surgery, Longhui County People’s Hospital, Longhui, 422200 Hunan China; 5 Nantong Haimen Yuelai Health Centre, Haimen, 226100 China; 6 Department of Respiratory, Nantong Traditional Chinese Medicine Hospital, Nantong, 226019 Jiangsu Province China; 7 Department of General Surgery, Haian people’s Hospital, Haian, 226600 Jiangsu China

**Keywords:** Epidemiology, HCC, ceRNA, ceRNET, Mechanism, Function

## Abstract

Hepatocellular carcinoma (HCC) is one of the leading causes of cancer-related death worldwide due to its high degree of malignancy, high incidence, and low survival rate. However, the underlying mechanisms of hepatocarcinogenesis remain unclear. Long non coding RNA (lncRNA) has been shown as a novel type of RNA. lncRNA by acting as ceRNA can participate in various biological processes of HCC cells, such as tumor cell proliferation, migration, invasion, apoptosis and drug resistance by regulating downstream target gene expression and cancer-related signaling pathways. Meanwhile, lncRNA can predict the efficacy of treatment strategies for HCC and serve as a potential target for the diagnosis and treatment of HCC. Therefore, lncRNA serving as ceRNA may become a vital candidate biomarker for clinical diagnosis and treatment. In this review, the epidemiology of HCC, including morbidity, mortality, regional distribution, risk factors, and current treatment advances, was briefly discussed, and some biological functions of lncRNA in HCC were summarized with emphasis on the molecular mechanism and clinical application of lncRNA-mediated ceRNA regulatory network in HCC. This paper can contribute to the better understanding of the mechanism of the influence of lncRNA-mediated ceRNA networks (ceRNETs) on HCC and provide directions and strategies for future studies.

## Current epidemiology of HCC

**Fig. 1:**
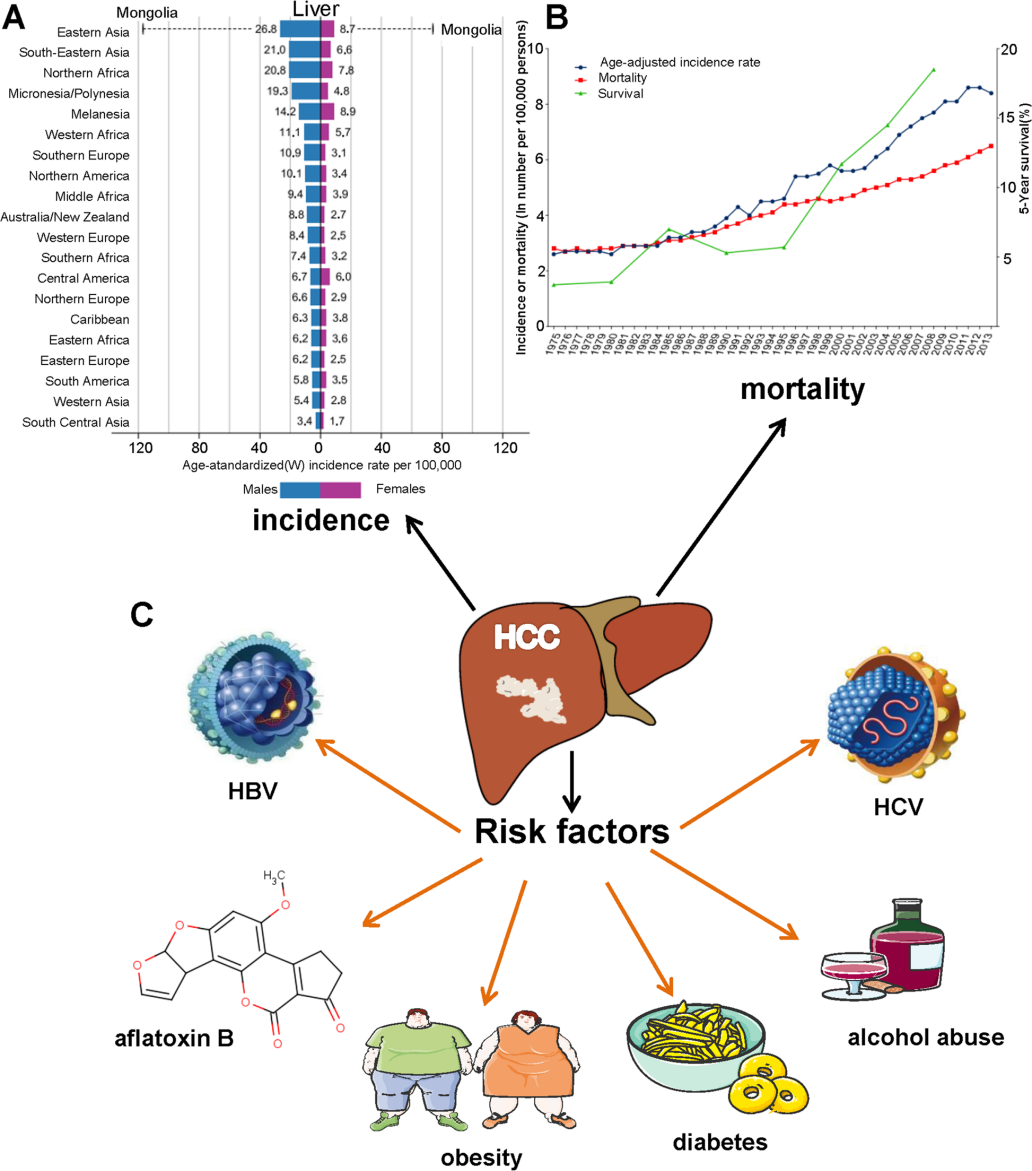
Incidence and risk factors of HCC. **A** Bar graph of Region-adjusted incidence of HCC (per 100, 000) worldwide in 2018, stratified by Sex. **B** Age - specific incidence and mortality rates (per 100, 000), and 5-year relative survival for HCC in the US from 1975 to 2013. **C** Risk factors: HBV, HCV obesity, aflatoxin B, diabetes, and alcohol abuse

Liver cancer, primarily hepatocellular carcinoma (HCC) [1], is the sixth most common cancer type and the fourth leading cause of cancer deaths worldwide. It was estimated in 2018 that there were 782, 000 deaths and 841, 000 new cases of liver cancer throughout the world, with a higher incidence in men than in women in many parts of the world [2, 3] (Fig. 1A). Moreover, the incidence and mortality rates of HCC vary widely across the globe (Fig. 1B), with the highest incidence rates in Eastern Asia and sub-Saharan Africa [4], followed by Southern Europe and lower rates in Europe and some regions of America [5].

The etiologic factors of liver cancer are very complicated. The leading causes of the HCC with high incidence include chronic hepatitis B virus infections (HBV), hepatitis C virus (HCV) infections, and food intake contaminated with aflatoxin B (Fig. 1C) [6–9]. Other risk factors include chemical carcinogens exposure, obesity, diabetes, and alcohol abuse (Fig. 1C) [10–13], and the HCC incidence has been on the rise in recent years. Meanwhile, there are significant geographical differences in the risk factors for HCC, mirroring the variation in global distribution patterns. In Asia (notably China) and Africa, the chronic HBV infection with high prevalence has caused an increase in the incidence rates of HCC [14]. In addition, HCV infection with high incidence has become the leading virus-related cause of HCC in some countries, such as Singapore, Japan, Australia, Europe, and the United States of America [15].

Statistics indicate that 5-years survival for HCC is 18%, suggesting that only 30–40% of patients are diagnosed in the early stage [16]. Curative-intent treatments for early-stage HCC include surgical resection, ablation, liver transplantation (LT) and transarterial chemoembolization (TACE) [17]. However, HCC is characterized by a high recurrence and metastasis rate, low detection rate for the curable stages, and ineffective therapeutic options [18]. The incidence and mortality of HCC that is accepted as cancer with poor prognosis and reduced survival are still on the rise. Hence, effective treatment strategies to improve the diagnosis, prevention, and treatment ability of HCC are of urgency.

In recent years, a new and promising molecular-targeted therapy based on the carcinogenic mechanism of HCC has been applied in clinical treatment. Proper understanding of the molecular mechanism of hepatocarcinogenesis and identification of target molecules and signal pathways related to tumor phenotype are essential for designing better treatments and preventing the disease [19]. Noncoding RNA (ncRNA), an RNA molecule that is widely expressed in organisms and cannot encode proteins, has been found to mediate normal cell processes and to be involved in various human diseases, including cancer [20–23].

## The emerging roles of competing endogenous RNA (ceRNA) in HCC

Noncoding RNA (ncRNA) can be divided into two major categories: long ncRNA and short ncRNA according to whether the length is greater than 200 nucleotides. It can also be subdivided into long no coding RNA (lncRNA), circular RNA (circRNA), transfer RNA (tRNA), ribosomal RNA (rRNA), microRNA (miRNA), small interfering RNA (siRNA), and small nucleolar RNA (snoRNA) [24–27], etc. Among them, lncRNA and miRNA mainly mediate post-transcriptional regulation, the dysregulation of which was found associated with many malignant tumors [28].

Long noncoding RNA (lncRNA) is a subtype of ncRNA with a transcriptional length of more than 200 nucleotides and was once considered to be unable to encode proteins [29]. However, with the development of research, it was discovered that a small amount of lncRNA could encode polypeptides. Numerous identified lncRNAs are transcribed by RNA polymerase II. They may be 5′-capped and polyadenylated, usually located in the nuclear and cytoplasmic [30–32]. The lncRNA-related data have been reported and annotated in LncBase and other databases. Compared with protein-coding genes, lncRNAs can be transcribed from almost every site in multiple genomes in transcription directions.

**Fig. 2:**
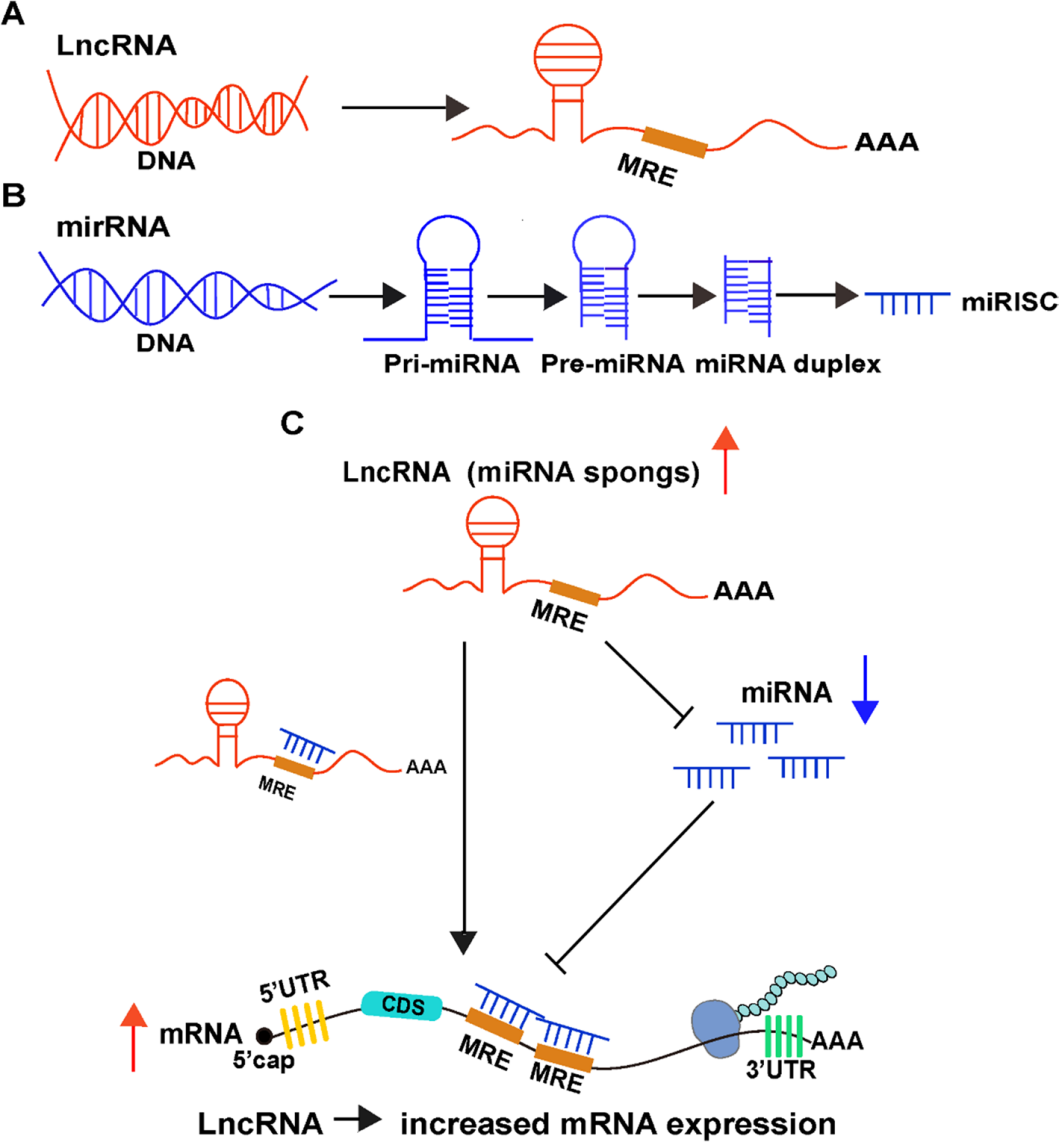
The mechanisms of lncRNA related ceRNA regulatory networks. **A**-**B** Biogenesis of long noncoding RNA (lncRNA) and microRNA (miRNA). **C** lncRNAs as competing endogenous RNAs and mRNAs share a pool of miRNAs, and the competition for miRNAs leads to dynamic regulation of the expression level of mRNAs

The ceRNA functions via a novel interaction mechanism between RNAs instead of deoxyribonucleic acid. In addition to directly regulating target genes, lncRNA can act as a miRNA sponge to bind miRNAs competitively [33], as suggested by the ceRNA hypothesis established in 2011, thereby weakening the inhibition of miRNA on target gene mRNA, indirectly improving the expression level of target genes, and regulating biological functions without being affected by protein translation (Fig. 2). Hence, these lncRNAs are known as competitive endogenous RNA (ceRNA).

Studies have shown that ceRNA, especially lncRNA are involved in all aspects of cancer. However, these long noncoding RNAs (lncRNA) have been considered as “junk genes” [34], while the functions of lncRNAs are found complex and diverse, which are characterized by biological regulation and explicit evolutionary conservatism [35].

Additionally, lncRNAs show more cell type-specific expression patterns than protein-coding genes, which supports the hypothesis that lncRNA is of functional importance. LncRNAs can be used as the organizational framework of subcellular structure to regulate protein localization or activity, and some lncRNAs can regulate global or local gene expression in trans or cis by influencing RNA polymerase II recruitment or inducing chromatin remodeling [36].

LncRNA can regulate gene expression through multiple mechanisms, such as directing chromatin remodeling complexes to specific sites, mediating mammalian X chromosome inactivation, and regulating DNA methylation and histone modification to induce epigenetic modification of DNA [37]. At the biological level, Different lncRNAs can mediate gene expression at the transcriptional, post-transcriptional and epigenetic levels.

Moreover, lncRNA is also involved in tumor biological processes, such as tumor proliferation, invasion, and metastasis. It has been reported that the dysregulated expression of lncRNA that can act as an oncogene or tumor suppressor gene may cause various cancers [38–40].

## The lncRNA-mediated ceRNA networks (ceRNETs) functions by modulating signaling pathways in HCC

**Fig. 3:**
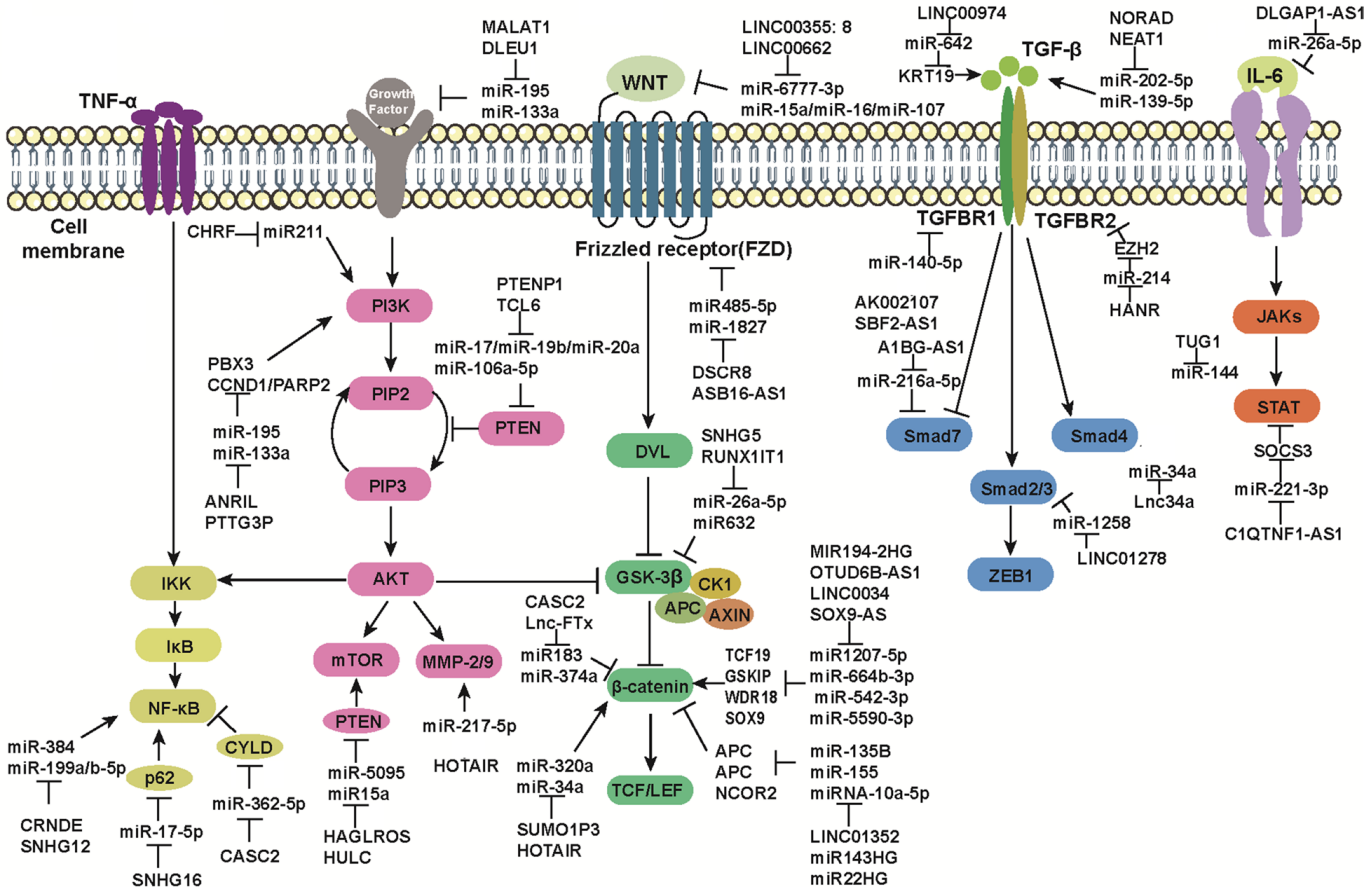
Schematic representation of five major signaling pathways. Schematic representation of five major signaling pathways involved in lncRNAs related ceRNETs regulation in HCC

More attention has been paid to molecular mechanisms that play a key role in the development of HCC. It has been observed that many cell signaling pathways are essential in regulating critical cell processes, such as cell proliferation, cell cycle progression, and apoptosis [41–43]. As a ceRNA, LncRNA also participates in HCC cell proliferation, epithelial to mesenchymal transition (EMT), invasion and metastasis by regulating signaling pathways in HCC, such as nuclear factor κB (NF-kB) pathway, phosphatidylinositol 3′-kinase(PI3K)-Akt signaling pathway, Wnt/β-catenin pathway, TGF-β pathway, and Janus kinase signal transducer and activator of transcription (JAK/STAT) pathway, which are very important in the process of carcinogenesis (Fig. 3).

### NF-κB pathway

NF-kB is a critical eukaryotic transcription regulator of inflammation [44]. Abnormal activation of NF-kB pathway often occurs in the early stage of HCC and is associated with the growth and invasion of HCC and EMT in HCC [45]. It was found that the lncRNA-mediated ceRNA networks can play biological functions by regulating NF-kB pathway. Oncogenic lncRNAs can exert the part of ceRNAs by activating NF-κB signaling in HCC. For instance, lncRNA SNHG16 can regulate p62 expression by sponging miR-17-5p, thereby mediating the NF-κB signaling pathway to promote proliferation, migration, and invasion of HCC cells and inhibit apoptosis [46]. LncRNA CRNDE can accelerate the progression of HCC by acting as the ceRNA of miR-384 to activate the NF-κB pathway [47]. LncRNA SNHG12 can promote the expression of MLK3 by competitively binding miR-199a/b-5p, thus activating the NF-κB pathway to promote the initiation and metastasis of HCC [48].

On the contrary, tumor suppressor lncRNAs acting as ceRNA may perform their biological function by inactivating the NF-κB pathway in HCC. For example, lncRNA CASC2, an important inhibitor of HCC, can up-regulate the expression of CYLD by competitively binding miR-362-5p, thereby inhibiting the NF-κB pathway to promote the growth and metastasis of HCC [49]. In conclusion, lncRNA-miRNA-NF-κB regulation network is expected to be a potential therapeutic target for HCC treatment.

### PI3K/AKT pathway

In nearly half of cases of HCC, the PI3K/AKT pathway was highly activated and was involved in critical cellular processes such as cell growth, survival regulation, and metabolism [50]. It has been reported that lncRNAs can play their biological functions by acting as miRNA ceRNAs to mediate the PI3K/Akt pathway. Among them, oncogenic lncRNAs by acting as ceRNAs can perform their biological effects by activating the PI3K/AKT pathway in HCC.

For example, lncRNA ANRIL can up-regulate the expression of PBX3 by acting as the ceRNA of miR-144, thereby activating the PI3K/AKT signaling pathway to promote the HCC growth, migration, and invasion, or inhibit apoptosis of HCC cells [51]. LncRNA CHRF and HOTAIR can activate the PI3K/AKT pathway and PI3K/AKT/MMP-2/9 pathway by sponging miR-211 and miR-217-5p, respectively, thereby promoting the viability, proliferation, and EMT process of HCC cells [52]. LncRNA PTTG3P can promote the expression of CCND1 and PARP2 by competitively binding miR-383, thus affecting the PI3K/AKT signaling pathway to promote the growth and metastasis of HCC cells [53]. LncRNA MALAT1 and DLEU1 can promote the expression of EGFR and IGF-1R by acting as the ceRNA of miR-195 and miR-133a, respectively, thereby activating the downstream PI3K/Akt signaling pathway to accelerate the HCC progression [54]. The expression of PTEN was reported to be decreased in nearly half of all HCC tumors, leading to the activation of the PI3K/Akt/mTOR pathway to promote HCC tumorigenesis. LncRNA HAGLROS and HULC can up-regulate the expression of ATG12 and P62 by sponging miR-5095 and miR15a, respectively, thereby affecting the PTEN/PI3K/AKT/mTOR signaling pathway to promote HCC cell survival and inhibit apoptosis [55].

However, tumor suppressor lncRNAs can exert the function of ceRNAs by inactivating PI3K/AKT or PI3K/AKT/mTOR in HCC. For example, lncRNA PTENP1 can up-regulate the expression of PTEN and other target genes by acting as ceRNAs of miR-17, miR-19b, and miR-20a, thereby inactivating PI3K/Akt/mTOR pathway to inhibit the HCC growth, proliferation, and migration and induce apoptosis of HCC cells [56]. LncRNA TCL6, a tumor suppressor lncRNA, can promote PTEN protein level by sponging miR-106a-5p, thus negatively regulating the PI3K/Akt signaling pathway in HCC to inhibit the HCC proliferation, migration, and invasion [57]. Therefore, lncRNA-mediated ceRNA networks can play their biological functions in HCC by mediating PI3K/AKT or PI3K/AKT/mTOR pathways and serve as promising targets for HCC treatment.

### Wnt/β-catenin pathway

Abnormal activation of Wnt/β-catenin signaling pathway is critical in the initiation and development of cancer. The elucidation on the regulatory mechanism of Wnt/β-Catenin pathway can provide new insights into anti-cancer therapy. lncRNAs can act as miRNA sponges by mediating the Wnt/β-catenin pathway in HCC to regulate the HCC progression, as supported by more studies.

Oncogenic lncRNAs can function as ceRNAs by activating Wnt/β-Catenin signaling in HCC. LncRNA SUMO1P3 can activate the Wnt/β-catenin pathway by inhibiting miR-320a, thereby accelerating the aggressive progression of HCC [58]. LncRNA LINC00355: 8 and SNHG5 can promote the expression of Wnt10b and GSK3β by sponging miR-6777-3p and miR-26a-5p, respectively, thereby activating the Wnt/β-catenin signaling pathway to promote HCC growth and EMT [59]. LncRNA LINC00662 can promote the expression and secretion of WNT3a by sponging miR-15a, miR-16, and miR-107, thus activating the Wnt/β-catenin signal transduction pathway to accelerate the HCC progression [60]. LncRNA HOTAIR can enhance the Wnt/β-catenin signaling pathway by down-regulating miR-34a, thereby promoting the drug resistance of HCC to paclitaxel [61]. LncRNA MIR194-2HG and OTUD6B-AS1 can up-regulate the expression of Transcription Factor 19 (TCF19) and GSK3B Interacting Protein (GSKIP) by competitively binding miR1207-5p and miR-664b-3p, respectively, thereby activating the Wnt/β-catenin signaling pathway and further promoting the proliferation and invasion of HCC cells [62]. LncRNA LINC00346 and SOX9-AS can promote the expression of WDR18 and SOX9 by acting as the ceRNAs of miR-542-3p and miR-5590-3p, respectively, thereby both regulating the Wnt/β-catenin pathway to promote the proliferation and metastasis of hepatoma cells [63]. LncRNA ASB16-AS1 and DSCR8 can up-regulate the expression of Frizzled Class Receptor 4 (FZD4) and FZD7 by sponging miR-1827 and miR485-5p, respectively, thus activating Wnt/β-catenin pathway and promoting the development of HCC [64].

However, tumor-suppressor lncRNAs that can act as ceRNAs, can perform their biological functions by inactivating the Wnt/β-catenin pathway in HCC. LncRNA LINC01352 directly binds to miR-135B and acts as a competitive endogenous RNA to promote the expression of miR-135B target gene APC Regulator of WNT Signaling Pathway (APC), thereby negatively regulating Wnt/β-catenin signaling to inhibit liver tumor development [65]. LncRNA RUNX1-IT1 can promote the expression of Glycogen Synthase Kinase 3 Beta (GSK3B) by competitively binding miR632, thereby inactivating the Wnt/β-catenin pathway of hepatoma cells to inhibit the proliferation, migration, and differentiation of cancer stem cells [66]. LncRNA CASC2 and Lnc-FTx can inactivate the Wnt/β-catenin pathway by down-regulating miR183 and miR-374a, respectively, thereby inhibiting EMT and invasion of HCC cells [67]. LncRNA MIR143HG and MIR22HG can act as ceRNAs of miR-155 and miRNA-10a-5p to up-regulate the expression of APC and Nuclear Receptor Corepressor 2 (NCOR2), thereby negatively regulating the Wnt/β-catenin pathway to inhibit the proliferation and metastasis of HCC [68]. In conclusion, the lncRNA-miRNA-Wnt/β-catenin regulatory network can provide rationales and insights for novel strategies of HCC treatment.

### TGF-β pathway

Studies have found the critical role of TGF-β signaling pathway in regulating cell growth, apoptosis, and differentiation, which also participates in the tumorigenesis and metastasis of HCC [69–72]. More importantly, lncRNA can regulate the progress of HCC by mediating the TGF-β signal pathway in HCC to play the role of miRNA sponge. Among them, oncogenic lncRNAs as ceRNAs can exert their biological function by activating the TGF-β signal pathway in HCC [73]. LncRNA NORAD can activate the TGF-β pathway by acting as the ceRNA of miR-202-5p and removing inhibitory effect of miR-202-5p on TGFBR1 and TGFBR2, thereby promoting the migration and invasion of HCC cells [74]. LncRNA LINC01278 and Lnc34a can up-regulate the expression of SMAD Family Member 4 (SMAD4) and SMAD2/3 by competitively binding miR-1258 and miR-34a, respectively, thereby activating TGF-β signaling to promote bone metastasis of HCC [75]. LncRNA AK002107 and SBF2-AS1 both target Transforming Growth Factor Beta Receptor 1 (TGFBR1) by sponging miR-140-5p, thereby promoting migration and invasion of hepatoma cells [76]. LncRNA HANR can up-regulate the expression of Enhancer of Zeste 2 Polycomb Repressive Complex 2 Subunit (EZH2) by competitively binding miR-214, thereby affecting the level of TGFBR2 to promote the HCC progression [77]. LncRNA NEAT1 can up-regulate the expression of TGF-β1 by competitively binding hsa-miR-139-5p, thus inducing the progression of HCC [78]. LncRNA HULC can inhibit Zinc Finger E-Box Binding Homeobox 1 (ZEB1) by competitively binding miR-200a, thereby promoting HCC progression and EMT [79]. LncRNA LINC00974 can promote the expression of Keratin 19 (KRT19) by sponging miR-642, thereby activating the TGF-β signaling pathway to expedite the proliferation and invasion of HCC [80].

On the contrary, tumor suppressor lncRNAs, by acting as ceRNAs, can exert its biological function by inactivating the TGF-β1 pathway in HCC [81–83]. For example, lncRNA A1BG-AS1 can promote the expression of Smad7 by acting as the ceRNA of miR-216a-5p, thereby inhibiting the proliferation and invasion of HCC [84]. Therefore, the lncRNA-mediated ceRNA network by mediating the TGF-β pathway can provide novel ideas for HCC treatment.

### JAKs/STAT pathway

The JAK/STAT pathway can be activated by multiple cytokines that is involved in many important cellular processes, such as cell differentiation, proliferation, and apoptosis [85–88]. The abnormal activation of the JAK/STAT signal can cause tumor cell migration and invasion in HCC [89]. Moreover, the lncRNA-mediated ceRNA network can exert its biological functions by regulating the JAK/STAT pathway, in which lncRNA, acting as ceRNA, can perform its biological function by regulating the JAK/STAT pathway in HCC. For example, lncRNA DLGAP1-AS1 can increase the level of oncogenic cytokine IL-6 by competitively binding miR-26a-5p, thereby promoting the occurrence and EMT of HCC through the JAK2/STAT3 signaling pathway [90]. LncRNA TUG1 can activate the JAK2/STAT3 pathway by inhibiting miR-144, thereby promoting the proliferation and migration of HCC cells [91]. On the contrary, the tumor suppressor lncRNA C1QTNF1-AS1 can up-regulate the Suppressor of Cytokine Signaling 3 (SOCS3) by sponging miR-221-3p, thereby inhibiting the proliferation, migration, and invasion of HCC cells through the JAK/STAT signaling pathway [92]. Therefore, lncRNA-miRNA-JAK/STAT regulatory network is a promising therapeutic target for HCC, which is expected to improve clinical efficacy as a personalized therapy.

## Clinical application potential of lncRNA-mediated ceRNET in HCC

**Fig. 4:**
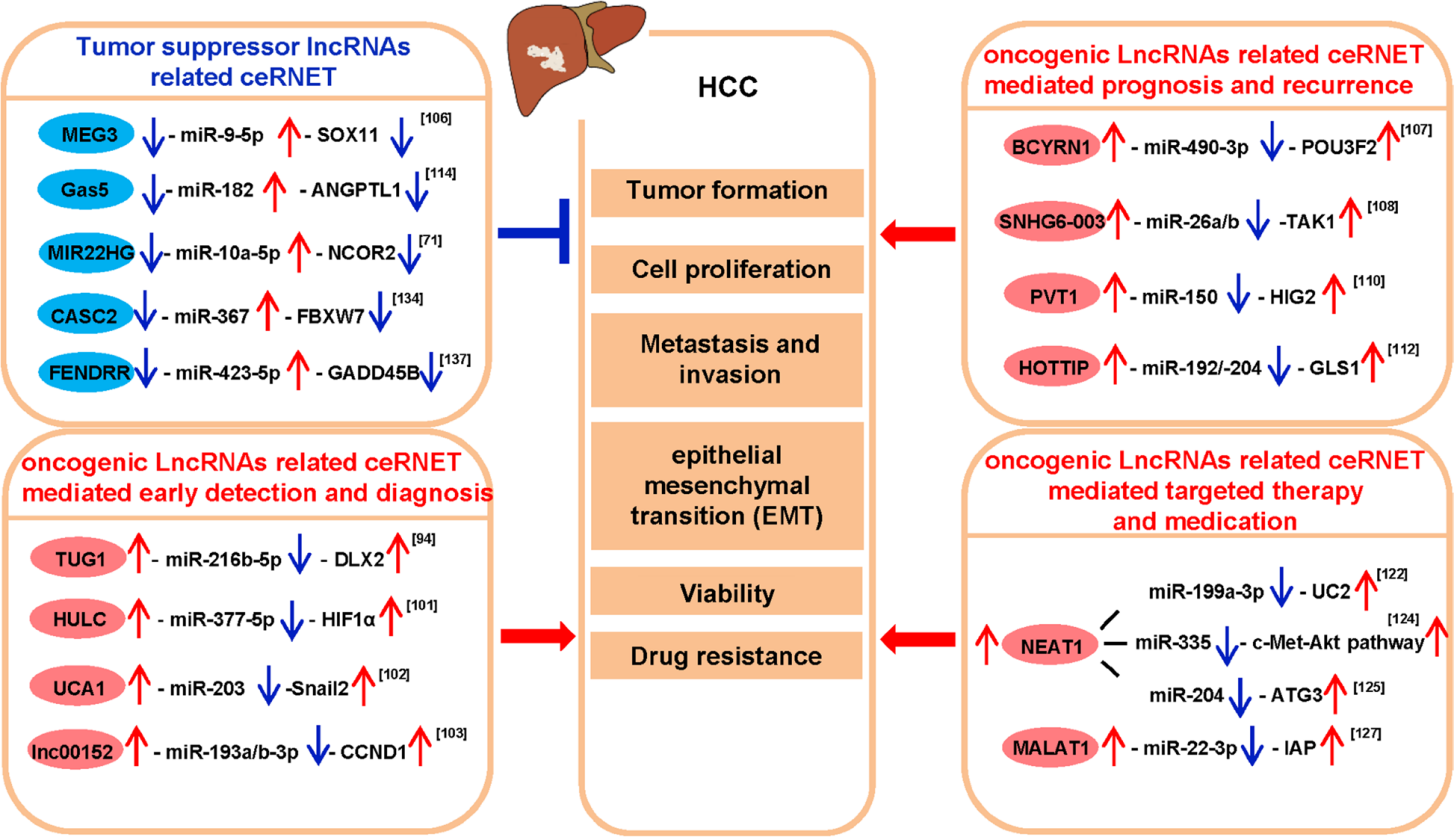
Summary of lncRNAs related ceRNETs mediated functions in HCC. LncRNAs can regulate HCC progressions, such as tumor formation, cell proliferation, metastasis and migration, epithelial–mesenchymal transition (EMT), and drug resistance

Currently, the most common detection methods for HCC are alpha-fetoprotein (AFP) detection and imaging examination. AFP is a conventional tumor biomarker for the clinical diagnosis of HCC. However, AFP has low sensitivity and insufficient specificity in the diagnosis of HCC, failing to detect about 30% of patients with early-stage HCC [93]. Liver ultrasound is effective in early tumor diagnosis and treatment, but its effectiveness in clinical practice needs to be improved [94]. Meanwhile, among the anti-cancer drug regimens for HCC, the first-line treatments such as sorafenib and lenvatinib can only prolong a short survival time for patients with advanced HCC [95]. Therefore, the search for new biomarkers to improve the diagnosis rate of HCC at an early stage is of urgency. A substantial body of evidence has demonstrated that lncRNA can act as ceRNA and can be used as biomarkers or even treatment targets for HCC (Fig. 4). Therefore, further studies on lncRNA may provide novel insights into the detection and diagnosis, prognosis and recurrence, and targeted medication and treatment of HCC.

## LncRNA related ceRNET mediated early detection and diagnosis potential in HCC

Although AFP is regarded as a gold standard biomarker in the detection and diagnosis of HCC, its sensitivity and specificity are insufficient, thus giving urgency of new biomarkers with better clinical significance. Several lncRNAs have been identified as ceRNAs in many cancers, including HCC. A growing body of evidence suggests that lncRNAs can act as ceRNA and related ceRNETs may play a significant role in clinical applications as diagnostic biomarkers for HCC.

Taurine Up-Regulated 1 (TUG1) has been proved to be a proto-oncogene, and the up-regulated TUG1 expression in the HCC group is correlated with the Barcelona Clinic Liver Cancer (BCLC) staging and tumor size compared with the control group [96]. Notably, the combined detection of TUG1 and AFP can improve the accuracy of HCC diagnosis [97]. Besides, TUG1 can enhance the expression of Distal-Less Homeobox 2 (DLX2) by acting as a ceRNA for miR-216b-5p, thereby promoting the growth and metastasis of HCC cells [98].

LINC00152 plays an oncogenic role in human malignancies, including HCC [99]. The expression levels of HULC and LINC00152 in HCC patients were marked higher than those in the control group, and the combined detection of HULC, LINC00152 and AFP enjoyed a high diagnostic sensitivity and specificity, with an Area under Curve (AUC) value of 0.89 [100]. Similarly, the expression levels of UCA1 and LINC00152 in HCC patients were significantly higher than those in patients with benign hepatopathy and healthy controls, and the combined detection of HULC, LINC00152 and AFP had even higher sensitivity and specificity of diagnosis, with AUC value of 0.912 and sensitivity and specificity of 82.9 and 88.2%, respectively [101]. HULC can promote the expression of Hypoxia Inducible Factor 1 Subunit Alpha (HIF1A) by sponging miR-377-5p, thus enhancing the proliferation and invasion of HepG2 cells [102]. Urothelial Cancer Associated 1 (UCA1) can activate the expression of transcription factor Snail2 by acting as the ceRNA of miR-203, thus promoting the proliferation, invasion, and EMT of hepatoma cells [103]. LINC00152 can promote the progression of the HCC cell cycle by sponging miR-193a/b-3p to regulate its target gene CCND1 [104]. In short, the three ceRNETs of HULC/miR-377-5p/HIF1α, UCA1-miR-203-Snail2 and lNC00152-miR-193a/b-3p-CCND1 are all promising targets for HCC detection and diagnosis.

However, some tumor suppressor genes have an active function in the HCC diagnosis. Maternally Expressed 3 (MEG3) is a tumor suppressor that is involved in the occurrence and development of various malignancies, including HCC. Both in vivo and in vitro experiments have confirmed that MEG3 can inhibit the proliferation and induce apoptosis of HCC cells, and Cox regression analysis found that MEG3 expression is an independent prognostic factor for HCC patients [105]. Meanwhile, MEG3 is a risk factor for elevated human AFP [106]. Therefore, MEG3 is a potential biomarker for predicting the prognosis of HCC. In addition, MEG3 can enhance SRY-Box Transcription Factor 11 (SOX11) expression by acting as a ceRNA for miR-9-5p, thereby inhibiting growth but promoting apoptosis of HCC cells [107]. Therefore, the ceRNET may provide new insights into the pathogenesis of HCC and provide a potential diagnostic marker for HCC.

On the other hand, technologies for assaying circRNAs have been greatly improved, but error and bias still affect detection accuracy. Theoretically, RNA-seq can identify circRNAs accurately within populations of different RNAs that constitute complete transcriptomes [108–111]. However, algorithms for analyzing the next-generation sequencing data often apply different problem-solving rules to minimize false positives and false negatives, leading to incorrect discoveries and misinterpretation in data.

Other variables that may confound interpretation of data include the introduction of sequence artifacts during library preparation, sequence-specific influences on splicing, biases resulting from rarity of circRNAs and difficulties with classifying splice junctions as true positives. Enriching circRNA populations by treating extracts with RNase R to eliminate linear RNAs is now widely used in studies employing microarray assays [112]. Direct assay of the fractions of circularized transcripts that are derived from parental genes will assist with interpretations about regulatory functions.

Because of the implication with the etiology of HCC, circRNAs can be used as potential markers of the malignancy and targets of therapeutic intervention and therapeutic agents. The field is at a critical development stage, thus giving importance of the clarification of relevant studies reconciling disparate observations, standardization of methodology, and devising methods to exclude possible artifacts.

## LncRNA related ceRNET mediated prognosis and recurrence potential in HCC

Although liver transplantation (LT) and partial liver resection (LR) are effective treatments for HCC, the prognosis is not ideal due to metastasis and recurrence. Recent studies have shown that lncRNA as a ceRNA can predict postoperative recurrence and survival time of HCC patients.

Brain Cytoplasmic RNA 1 (BCYRN1) is found of prognostic value besides its diagnostic function in HCC. Overexpression of BCYRN1 can promote the growth and migration of HCC cells in vitro with being overexpressed in HCC samples. Therefore, BCYRN1 can serve as a prognostic marker for HCC [113]. Meanwhile, BCYRN1 can promote the expression of POU3F2 by sponging miR-490-3p, and the ceRNET mechanism is more significant in regulating the prognosis of HCC [114].

LncRNA SNHG6–003 can promote cell proliferation and induce drug resistance in HCC cells, and its high expression is correlated with the poor prognosis of HCC patients. SNHG6–003 can regulate the expression of transforming growth factor-β activated kinase 1 (TAK1) by acting as the ceRNA of miR-26a/b [115]. Therefore, targeted study on this ceRNET is expected to find a therapeutic strategy for HCC.

Human lncRNA-PVT1 expression can be up-regulated in HCC tissues, and patients with high expression of lncRNA-PVT1 had poor clinical prognosis. Human lncRNA-PVT1 can promote the proliferation, cell cycle and stem cell-like properties of HCC cells [116], as demonstrated by in vitro and in vivo experiments. Meanwhile, the up-regulation of miR-150 expression by knockout of PVT1 gene can reduce the expression of Hypoxia Inducible Gene 2 (HIG2) and inhibit the proliferation, invasion, and iron metabolism balance of HCC [117]. Therefore, this ceRNET will contribute to the study of the pathogenesis of HCC.

Multivariate analysis showed that the high expression of HOXA Distal Transcript Antisense RNA (HOTTIP) was an independent risk factor for tumor recurrence and shorter overall survival after LT. In vitro studies showed that down-regulation of HOTTIP expression could reduce the aggressiveness of tumor cells and increase their chemo-sensitivity. Therefore, the expression level of HOTTIP can be a predictor of tumor recurrence after LT in HCC patients [118]. In addition, the miR-192/− 204-HOTTIP axis may inhibit the viability of HCC cells by down-regulating GLS1, and the combined indicators of miR-192/− 204 and HOTTIP are more significantly correlated with the prognosis of HCC.

Nevertheless, some tumor suppressor genes are risk factors for poor prognosis of HCC. Multivariate analysis revealed that the up-regulation of lncRNA Gas5 expression was an independent predictor of poor recurrence free survival (RFS) (HR: 1.287, 95% CI: 1.027 ~ 1.612, *P* = 0.028). Methylation data showed that the methylation status of 5 CpG sites (cg07177756, cg17025683, cg16290996, cg03044573 and cg06644515) was moderately negatively correlated with the expression of Growth Arrest Specific 5 (Gas5) (*P* = − 0.54, *P* < 0.001). Therefore, Gas5 plays a vital role in HCC prognosis [119]. Moreover, Gas5 spongs miR-182 can up-regulate anti-metastatic protein Angiopoietin Like 1 (ANGPTL1) expression and then inhibit tumor metastasis, which can play a crucial role in the therapeutic intervention against the progression of HCC [120].

Another tumor suppressor gene is miR22HG. The silico and experimental analyses showed that overexpression of miR22HG could inhibit the proliferation, invasion, and metastasis of HCC cells, while low expression of miR22HG is related to tumor progression and poor prognosis of HCC patients. Therefore, miR22HG is a potential prognostic biomarker [121]. Moreover, miR22HG can suppress the activation of the Wnt/β-catenin pathway by sponging miR-10a-5p to up-regulate the expression of the target gene NCOR2, thereby inhibiting the progression of HCC [68]. In conclusion, ceRNET can provide new clues for the development of novel prognostic biomarkers for HCC patients.

## The potential of LncRNA related ceRNET as therapy target in HCC

Conventional cancer treatments are effective only in some patients with the major issue of drug resistance. Therefore, exploration of the potential mechanism of HCC resistance and effective drug-specific targets that target tumor cells without adversely affecting normal cells will help to improve the cure rate of HCC. LncRNA has been considered a potential biomarker candidate for targeted therapy and medication in cancer due to its higher specific expression in tumor cells, less side effects, and critical role in mediating resistance [122].

LncRNA NEAT1 plays a carcinogenic role in various tumors, including gastric cancer, lung cancer, colorectal cancer and liver cancer [123]. Recent studies have shown that NEAT1, as a ceRNA, can be a new target for HCC therapy. NEAT1 expression can be up-regulated in HCC tissues, and NEAT1 can promote the expression of Uridine-Cytidine Kinase 2 (UCK2) by competitively binding to the tumor suppressor miR-199a-3p, so as to maintain the growth of HCC cells [124]. More importantly, NEAT1-mediated ceRNET can affect the drug resistance of HCC. Studies showed that sorafenib resistance could be induced by inhibiting sorafenib induced apoptosis by activating the c-Met-Akt pathway in vivo and in vitro [125]. However, NEAT1 can enhance the drug resistance of HCC cells to sorafenib by sponging miR-335 to relieve the inhibition of the c-Met-Akt pathway [126]. Meanwhile, NEAT1 can also act as the ceRNA of miR-204 to up-regulate the expression of Autophagy Related 3 (ATG3), thus promoting HCC autophagy and enhancing the resistance of HCC cells to sorafenib [127].

Metastasis Associated Lung Adenocarcinoma Transcript 1 (MALAT1) is one of the most widely studied lncRNAs in cancer therapy. lncRNA MALAT1 was found to play an oncogenic role in the occurrence and development of HCC and act as a proto-oncogene to regulate the expression of apoptosis inhibitor IAPS [128]. MALAT1 can act as the ceRNA of miR-22-3p to cause the abnormal expression of the target gene inhibitor of apoptosis proteins (IAPs) [129]. Meanwhile, MALAT1 is involved in BA-induced apoptosis in HCC, and betulinic acid (BA) can inhibit the expression of IAPs and MALAT1 in HCC. BA is a natural drug extracted from various Chinese herbal medicines [130] with satisfactory anti-cancer activity, selectively cytotoxic to cancer cells but not to normal cells, which can induce autophagy and apoptosis of cancer cells [131]. BA can affect IAP in HCC by activating miR-22-3p to target MALAT1, thus inducing the death of HCC cells [132]. Therefore, MALAT1 is a potential biomarker involved in the drug treatment mechanism of HCC.

Similarly, some lncRNAs also play an essential role in the treatment of HCC as tumor suppressor factors. Cancer Susceptibility 2 (CASC2) is a useful liver cancer cell regulator whose expression can be down-regulated in multiple cancers, including HCC [133]. Overexpression of CASC2 can promote the apoptosis of HCC cells and inhibit cell proliferation and metastasis [134]. In addition, CASC2 can promote F-Box and WD Repeat Domain Containing 7 (FBXW7) by sponging miR-367, thereby inhibiting EMT, migration, and invasion of HCC cells [135]. Meanwhile, CASC2 plays a key role in the drug resistance of HCC. CASC2 can improve the sensitivity of HCC cells to cisplatin (DDP) by downregulating the expression of miR-222, which provides a promising therapeutic strategy for overcoming DDP resistance in HCC [136].

FOXF1 Adjacent Non-Coding Developmental Regulatory RNA (FENDRR) is related to the progression of various tumors. In HCC, FENDRR can inhibit the growth and metastasis of HCC cells by down-regulating Glypican 3 (GPC3) expression [137]. FENDRR can also act as the ceRNA of miR-423-5p to up-regulate Growth Arrest and DNA Damage Inducible Beta (GADD45B), inhibit HCC growth, promote the apoptosis of HCC cells, and regulate the immune escape of HCC mediated by Tregs. Therefore, ceRNET associated with FENDRR is a new therapeutic target worth consideration.

## Outlook

HCC is one of the most complex cancers with increasing incidence and mortality rate and various potential pathogenic mechanisms. Many risk factors contribute to the initiation of HCC, and the rates of metastasis and recurrence of HCC remain high, giving huge challenges to the current treatment strategies. So far, an increasing number of preclinical studies showed that lncRNA is a good diagnostic and prognostic biomarker for HCC treatment. Many lncRNAs can influence the cellular biological processes of HCC, such as cell proliferation, invasion, migration, apoptosis, and EMT, by competitively binding miRNAs of ceRNA and mRNA, which lays a foundation for promising clinical indicators for early detection, diagnosis, prognosis, recurrence, therapy and targeted medication of HCC.

However, studies on the detailed mechanism of the ceRNA network and its relationship with HCC are still in the initial stage. Although increasing attention has been paid to lncRNA as ceRNA in HCC, less was to the interaction between lncRNA-mediated ceRNA regulatory networks in HCC. Therefore, it is necessary to study the identity, role, and mechanism of ceRNAs in different malignant stages of HCC. To date, studies on ncRNAs that act as ceRNAs in HCC have primarily involved overexpression and knockout assays in cells and animals.

Moreover, other factors can affect ceRNA activity, such as subcellular location and ceRNA component abundance, interactions with RNA binding proteins, RNA editing, and ceRNA affinity in the endogenous cellular context. However, whether the results of overexpression assays can truly reflect the spontaneous ceRNA crosstalk during carcinogenesis in patients with HCC remains unknown. Therefore, more animal experiments and clinical trials should be conducted for verification.

Additionally, the majority of identified ceRNA interactions show single binding partners, although ceRNA crosstalk in large interconnected networks. Apart from direct interactions via shared miRNAs, secondary and indirect interactions might also significantly affect ceRNA modulation.

Therefore, further investigations on ceRNAs should focus not only on identifying binary ceRNA interactions but on the network analysis of potential complex miRNA and ceRNA networks. Moreover, the scale-free network property of ceRNA regulation also poses a challenge when selecting HCC-related molecular therapeutic targets. Targeting nonessential nodes within regulatory networks could cause ineffective therapeutic responses, as cancer cells may overcome the resulting damage through alternative signaling pathways. Therefore, therapeutic targets situated in a hub position of a ceRNET should be considered in future screening studies for HCC therapeutic targets.

In conclusion, the recently developed research techniques and computational methods will support a more comprehensive study on lncRNA that acts as ceRNA and mediated ceRNET in HCC. These findings can not only contribute to a better understanding of the pathogenesis and progress of HCC but also help to create new directions and strategies for the prevention, diagnosis, and treatment of HCC.

## Data Availability

The data that support the findings of this study are available from the corresponding author upon reasonable request.
